# Laser-initiated primary and secondary nuclear reactions in Boron-Nitride

**DOI:** 10.1038/srep21202

**Published:** 2016-02-17

**Authors:** C. Labaune, C. Baccou, V. Yahia, C. Neuville, J. Rafelski

**Affiliations:** 1 LULI, Ecole Polytechnique, CNRS, CEA, UPMC, 91128 Palaiseau, France; 2 The University of Arizona, Tucson, AZ 85721-0081, USA

## Abstract

Nuclear reactions initiated by laser-accelerated particle beams are a promising new approach to many applications, from medical radioisotopes to aneutronic energy production. We present results demonstrating the occurrence of secondary nuclear reactions, initiated by the primary nuclear reaction products, using multicomponent targets composed of either natural boron (B) or natural boron nitride (BN). The primary proton-boron reaction (p + ^11^B → 3 α + 8.7 MeV), is one of the most attractive aneutronic fusion reaction. We report radioactive decay signatures in targets irradiated at the Elfie laser facility by laser-accelerated particle beams which we interpret as due to secondary reactions induced by alpha (α) particles produced in the primary reactions. Use of a second nanosecond laser beam, adequately synchronized with the short laser pulse to produce a plasma target, further enhanced the reaction rates. High rates and chains of reactions are essential for most applications.

The proton-boron reaction is one of the most interesting candidates for fusion energy production as it produces less than 1% of the energy in neutrons, so it is called aneutronic fusion. However, it seems unreachable with conventional fusion approaches because it requires a very high temperature^1^, and consequently a very high laser energy, and it seems difficult to achieve a positive energy balance taking into account the nuclear energy release and the radiation losses^2^. Radically different approaches and schemes must be sought^3^,^4^. Taking advantage of the fast evolution of the short, high-intensity lasers^5^, we proposed a new scheme^6^ based on fusion reactions initiated by a laser-accelerated proton beam^7^,^8^ and we demonstrated its high efficiency compared to previous approaches^9^,^10^. Nonetheless, it is clear that it will not be possible to reach a positive energy balance without cycles of reactions to increase the overall fusion yield. The present work demonstrates the possibility of producing reactions chains initiated by charged particles using multi-component targets irradiated by laser-accelerated particles.

## Results and Discussion

### Nuclear pB, pN and secondary α–induced reactions

The proton-boron fusion reaction has been extensively studied and produces three alpha particles through three main channels^11^:


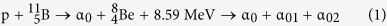



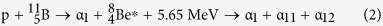



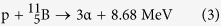


The predominant channels go through the formation of a compound nucleus ^12^C* followed by sequential α decays via an unbound ^8^Be in its fundamental (1) or first-excited (2) state. Channel (3) corresponds to the direct 3-body reaction that, for low-energy protons, contributes to less than 5% to the total cross-section^12^. The alpha particle spectrum that emerges derives from these reactions and is between a few hundred keV to ~10 MeV given the fusion yield and available proton energy^12^,^13^, with a strong wide peak near to 4 MeV, with location somewhat dependent on proton energy. A fourth γ capture fusion path exists where the ^12^C* compound nucleus decays by γ emission, releasing ~15.9 MeV, but in comparison this is a highly improbable reaction. The cross-section of the ^11^B(p,α)2α reaction as a function of the centre of mass energy is characterized by two low-energy resonances at E_c.m_ = 148 keV and E_c.m_ = 614 keV^11^,^14^, that are very interesting for producing high numbers of reactions.

**Figure 1:**
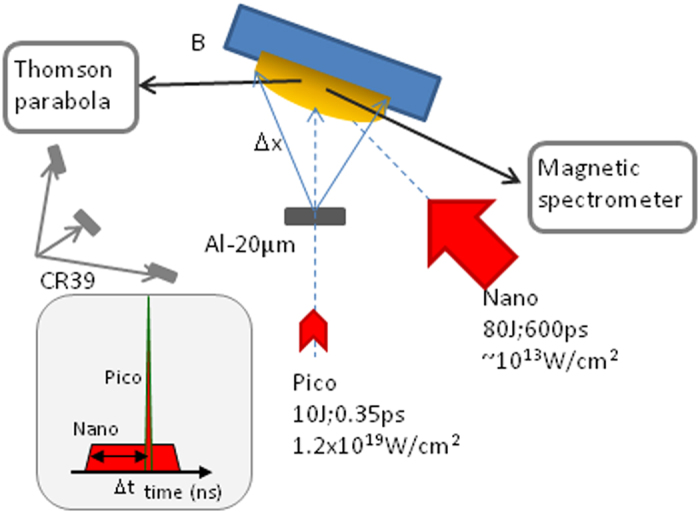
Scheme of the set-up to study interaction between a proton beam accelerated by a short, high-intensity laser pulse and a solid or plasma boron or boron-nitride target. The two laser pulses have a well-defined timing with a time-delay as shown in the insert. The particles diagnostics are displayed around the irradiated target.

Other nuclear reactions between the accelerated protons and the boron target can occur. In the experiments described in Methods, a schematic view of the set-up is shown in Fig. 1, we used natural boron that is comprised of two isotopes 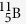 (80.1 ± 0.2%) and 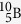 (19.9 ± 0.2%). The two other primary nuclear reactions of interest in this context are^15^,^16^:


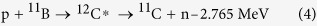



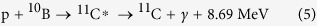


The cross sections for these two reactions reach significant values for proton energy above 3 and 1.5 MeV respectively. However, reaction (5) is ~one hundred times less probable than reaction (4). The radioactive ^11^C has a half-life of (20.364 ± 0.014) min by positron decay:


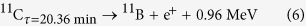


Finally, the low-energy protons can also interact with ^10^B to produce ^7^Be by the reaction:


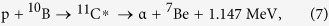


which produces alpha particles. ^7^Be decays via electron capture with a half-life of 53.24 days.

Secondary nuclear reactions can occur between the *in situ* produced α-particles, generated by the (1–3) reactions and ^10^B^17^,^18^:


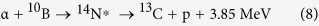


which regenerates high-energy protons, and


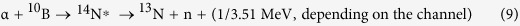


which requires alpha energy larger than 2.5 MeV to reach significant cross section values. The radioactive ^13^N has a half-life of 9.97 minutes by positron decay:


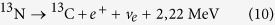


**Figure 2:**
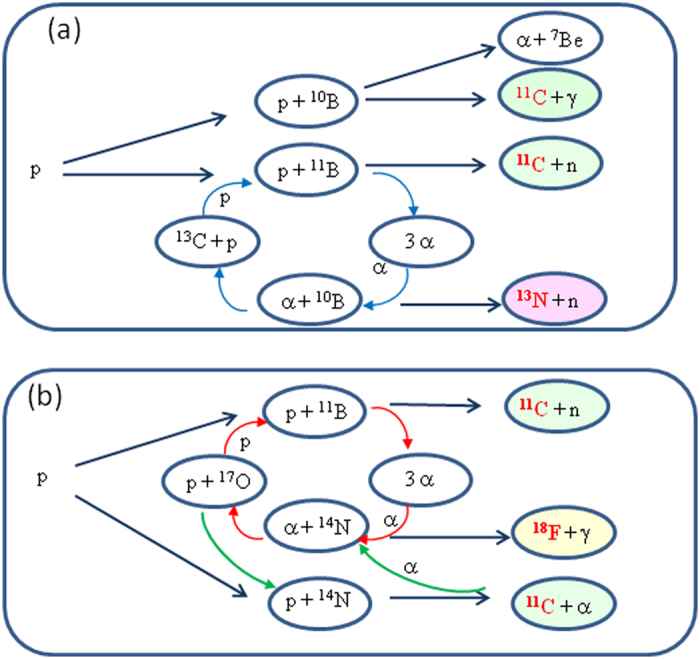
Scheme of the primary and secondary nuclear reactions produced by the interaction between a laser-accelerated proton beam and (**a**) a natural boron target, (**b**) a boron-nitride target. In the case of the BN targets the reactions with ^10^B can also occur but are not shown for clarity.

A synthetic schematic of the primary and secondary reactions of interest in our study, initiated by the interaction of protons irradiating a natural boron target, is shown in Fig. 2a.

The novel idea of these experiments was to use compound targets to take advantage of both the wide spectrum of energy and high intensity of the laser-accelerated proton beam to initiate chains of reactions based on complementary elements and of the secondary reactions driven by the alpha particles. Some of these are producing protons that promise the continuation of chain reactions and in principle the development of energy producing devices. We thus study a reaction cycle on a multicomponent, e.g. BA, target in which the primary reactions between the protons and the component B produces an α-particle which then reacts with the component A: p + B −>α + X, α + A −>p + Y.

We looked for an isotope that in reactions with α-particles produces protons and does not produce neutrons and in irradiation with either α-particles or protons does not go into undesirable side chains of reactions. A weakly (1–2 MeV) endothermic reaction is fine if this keeps up the chain of reactions. Allowing for endothermic reactions produced, we found one strongly aneutronic candidate, nitrogen, which further is easy to handle combined with boron. So we chose to use boron-nitride (BN) targets. Natural nitrogen is comprises of two isotopes 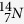 (99.636%) and 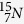 (0.364%). The primary and secondary nuclear reactions between the proton beam and the boron-nitride target are shown in Fig. 2b. The key α-induced secondary reaction of interest for our purpose is^19^,^20^:


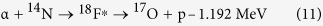


which produces energetic protons since the energy of the α -particles transfers to a large extend to the produced proton; low-energy α cannot react considering the relatively strong Coulomb repulsion. The cross section is indeed resonant and peaked at a few MeV, just where the fusion product α -particles appear, and therefore the protons produced are energetic.

The other α-induced secondary reaction^21^:


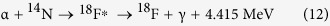


is of interest in medical applications given the radioactive ^18^F production. ^18^F has a half-life of 109.8 minutes by positron emission (97%) and by electron capture (3%) and will be used to diagnose this reaction.

Furthermore, ^11^C can also be produced by the following reaction:


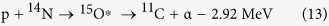


However to achieve an effective reaction rate of a few per 10,000 in solid target a proton energy larger than 6 MeV is needed^22^.

More nuclear reactions are possible in this context, but we do not consider them in a first approach because either their cross section is too small at the involved energies or the proportion of present elements is too small to contribute significantly to the observations.

### α-particles characterization

**Figure 3:**
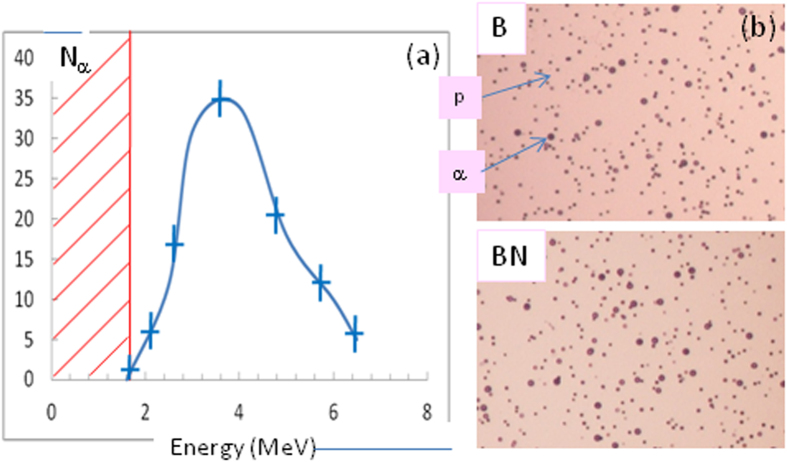
(**a**) Spectrum of the high-energy component of the α-particles produced by the interaction of a laser-accelerated proton beam and a boron target. The red line in the spectrum corresponds to the cutoff of the 6 μm Aluminum filter in the magnetic spectrometer. The error bars take into account the variation of the number of impacts across the direction perpendicular to the magnetic field. (**b**) CR39 images for B and BN targets showing the large impact diameter associated with the α-particles and the small impact diameters associated with protons.

The α-particles were observed and characterized with different diagnostics. An example of the high-energy spectrum measured in the magnetic spectrometer for a solid boron target is shown in Fig. 3a. The low-energy cutoff of the spectrum is due to the Aluminum filter with 6 μm thickness placed in front of the spectrometer. ~10^8 ^sr^−1^ α-particles with energy between 2 and 7 MeV are observed. Low-energy α-particles, between 500 and 900 keV, were observed in the Thomson parabola, but these particles are of less interest for the studies presented here.

α-particles were also observed with the boron-nitride targets. The spectrum of the high-energy particles is similar to that of the boron target but we observed a larger number of α-particles with BN both in the magnetic spectrometer and in the CR39 track detectors placed at different angles. These detectors were covered with 6 and 10 μm of Aluminum and so the observed particles have an energy higher than 2 and 3 MeV respectively. Typical images of CR39 covered with 6 μm of Aluminum for B and BN targets are shown in Fig. 3b.

### Activation measurements

**Figure 4:**
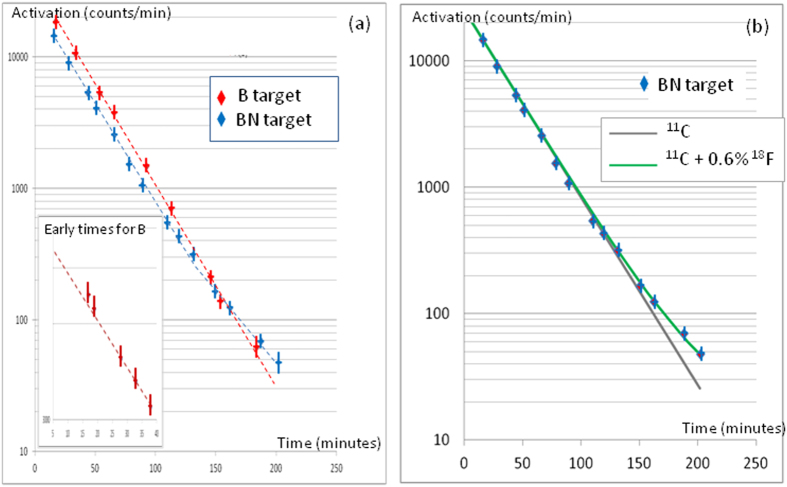
(**a**) Activation curves as a function of time for B and BN targets. The inset of part (**a**) is an enlargement of the beginning of the curve for the B target. (**b**) Fit of the BN activation curve using the sum of the contributions of ^11^C and ^18^F. A Geiger-Mueller tube detector with a precision of ±5% was used, performing a series of quick measurements comprising 4–16 readouts resulting in activity measurement error that was limited by the statistical count and timing width of about half to one minute. The final measurement error for the radio activity was thus in the range of 2–10% depending on yield and activity lifespan.

Examples of the time evolution of the normalized activation for B and BN targets are shown in Fig. 4. In both cases, the average slope in Logarithm scale, for t < 150 minutes, corresponds to the half-life of the ^11^C, (20.4 ± 0.2) minutes. A very interesting feature appears at late times in the BN curve. For t > 90 min, in the case of BN, we observe a clear reduction of the slope of the curve. This is not observed in the B target case. By analyzing the curve, we found that the slope at the latest times was close to 100 minutes. We attributed this change to ^18^F whose contribution begins to appear when ^11^C has already decreased in a significant way because the half-life of ^18^F is ~5 times longer than the half-life of ^11^C. We calculated a fit of this curve using the sum of the two exponentials,


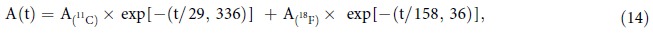


where 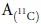 and 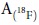 are the ^11^C and ^18^F activations respectively. In this particular case, we found a good fit for 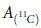 = 500 Bq and 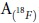 = 3.33 Bq, and interpreted the measured activation as the superposition of the ^11^C and ^18^F decays. This corresponds to ^11^C and ^18^F yields of 10^6^ and 6 × 10^3^ respectively. When analyzing more shots, we found that the ^18^F yields varied between <10^3^ (limit of detectability of our system) and 6 × 10^3^, which correspond to the error bars of our measurements.

At early times, t < 30 min, we observe that the activations measured in the experiment decrease faster, as a function of time, than the theoretical decay of ^11^C. This is shown in the insert of Fig. 4a. This must be due to the contribution of a short-lived component that, for our conditions, could be Nitrogen-13. We observed that its proportion was larger in the B than in the BN targets, but it was not possible with the actual precision of our instrumentation to deduce an accurate yield of ^13^N. This is due to the fact that the half-lives of ^13^N and ^11^C are too close and the access to the target takes ~14 minutes after the shot, so early essential measurements were not possible. Considering the possible nuclear reactions, the ^13^N is most likely produced by reaction (9), ^10^B(α,n)^13^N. As there are twice as many ^10^B in the B compared to the BN targets, this explains the observed higher ^13^N yields in B compared to BN targets.

**Figure 5:**
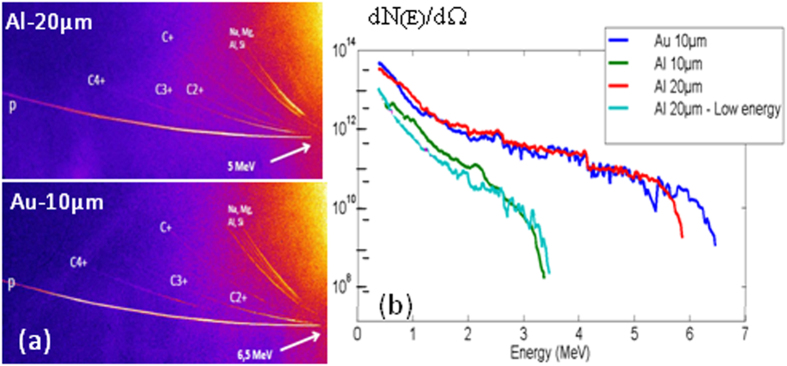
Characterization of the proton beam produced by the short, high-intensity (1–2 × 10^19 ^W/cm^2^) laser pulse with various targets using Thomson parabola. (**a**) Image of the Thomson parabola traces. (**b**) Integrated normalized proton flux per unit of solid angle, comprising all particles above energy E, as a function of the energy as deduced from the Thomson parabola analysis. The high-energy cutoff is modified by the conditions of interaction: nature of the target, intensity of the short pulse.

We made a few tests, either by changing the proton energy spectrum or the irradiated target, to unequivocally identify the involved nuclear reactions. The proton spectrum was modified by changing the thin foil (thickness or nature) irradiated by the picosecond beam or by varying the intensity of the pulse. We first consider reactions producing ^11^C in B targets. As a first test, we irradiated natural B targets with a proton beam accelerated out of an Aluminum foil of 10 μm thickness for which the maximum proton energy is ~3–3.4 MeV (see Fig. 5). The activation level was reduced by ~5 compared to the one obtained with a 20 μm Aluminum foil. A similar effect was observed by using Al-20 μm targets with a reduced intensity of the picosecond laser, whose main consequence was to remove the high-energy part of the proton spectrum but also to reduce the low-energy part. The activation level was then reduced by a factor ten. In both cases, as expected from its cross section^15^, the efficiency of the reaction ^11^B(p,n)^11^C was reduced, explaining the observed reduction of the activity. To check the efficiency of the ^10^B(p,γ)^11^C reaction, we irradiated ^10^B targets with protons produced by an Al-20 μm foil at maximum intensity. The total activation was reduced by ~60 compared to the one obtained with natural boron, demonstrating the very low efficiency of the reaction (5) compared to (4) in agreement with their respective cross sections.

### Modification of the ^11^C production in a plasma

An interesting part of the experiment was comparing the results from B and BN targets where both solid and plasma targets were explored. Typical maximum activation levels with solid B were ~(1000 ± 50) Bq, which correspond to ~2 × 10^6^ atoms of ^11^C at t = 0 (time of the shot). The contributions of ^13^N and ^18^F are considered as negligible in a first approach. In BN targets, for all shots in solid, the activation levels were below those of B targets by a factor ~2. Most likely, this result simply reflects the fact that the number of active atoms is reduced by a factor 2 in BN targets compared to B targets. This also confirms that the reaction (13) is ineffective in agreement with its threshold of 6 MeV and the energy spectrum of the laser-accelerated proton beam. So, most of the observed ^11^C was produced by reaction (4).

**Figure 6:**
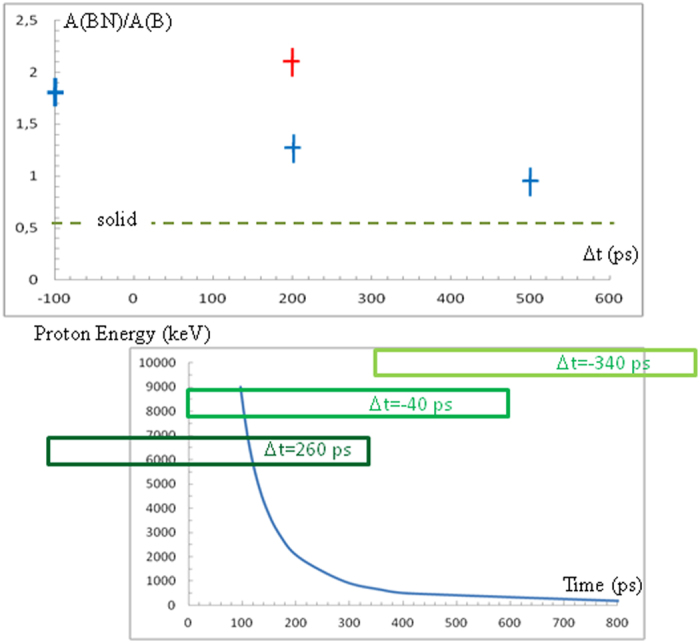
(**a**) Ratio of the activation extrapolated to t = 0 for a BN target, A(BN), compared to a B target, A(B), as a function of the time delay between the two laser pulses. A delay of Δt = −100 ps means that the short pulse was sent 100 ps before the long pulse. (**b**) Time of arrival of the protons on the target as a function of their energy for a distance of 6 mm between the two targets. The rectangles show the plasma forming pulses for the three time-delays.

The surprising result was the increase of activation in the case where the BN targets were conditioned in the plasma state by the nanosecond beam. From the temporal analysis, the activation corresponds again mainly to ^11^C production. In previous studies^6^, we already observed that the number of nuclear reactions was enhanced by plasma formation of the irradiated target. The interesting fact in this experiment is that the increase of activation is significantly greater in BN than in B plasmas. In Fig. 6a, the ratio of activation in BN versus B targets, A(BN)/A(B), is reported as a function of the time delay between the short and the long laser pulses. A delay of Δt = −100 ps means that the short pulse was sent 100 ps before the long pulse. Taking into account the distance of 6 mm between the two targets, this means that the most energetic protons arrive in the B/BN target when it is just beginning to be transformed into plasma. We observe that A(BN)/A(B) decreases from ~1.8 to 0.9 when Δt varies from −100 ps to +500 ps and is always larger in plasma than in solid where it is ~0.5. Using a gold-10 μm accelerated proton beam further increased, by ~50%, the ratio of ^11^C production in BN in comparaison to B plasmas. This latter result is likely due to the reaction (13) becoming active because of higher energy protons available with gold target.

## Discussion

Looking at the possible reactions in our experiment (Fig. 2), we interpret the observation of Fluorine-18 and Nitrogen-13 in the irradiated targets as the existence of secondary reactions driven by the α-particles produced by the primary reactions. In our experiments the α-particles are mainly produced by the ^11^B(p,α)2α, (1–3), which in turn react with ^14^N through the reaction (12), ^14^N(α,γ)^18^F, to produce the observed Fluorine-18 and with ^10^B through the reaction (9), ^10^B(α,n)^13^N to produce the observed Nitrogen-13. So the first step of our experimental proof of concept was successful, showing that it is possible to produce secondary reactions involving α-particle interaction with a second component of the target. In the same way, secondary protons are likely produced by reactions (8) and (11) with an energy large enough to induce new reactions with ^11^B.

In our BN targets, the observation of ^18^F radioactivity can be used as a diagnostic tool to estimate the yield of secondary protons produced in reaction (11): typically for each reaction of the type (12), where an observable radioactive isotope ^18^F is produced, there are up to 500 reactions (11) that produce an energetic proton. The exact ratio between (11) and (12) for *in situ* injected α -particle fusion spectrum in plasma environment where collective effects can be present is not known and could be further studied in future laser-plasma experiments. A similar diagnostic role is played by ^13^N activity. It is generated in reaction (9) which occurs also as a fraction of reaction (8)^18^. Additional experiments are needed to establish this ratio reliably, but one can presume that it is close to unity for the range of α-particle spectra available *in situ*.

In order to estimate the relative yield of ^11^C produced in primary proton-induced reactions as compared to secondary ^18^F and ^13^N we need to remember that effective production of ^11^C requires p at energy above 3 MeV while secondary α are products of pB fusion which occurs at much lower proton energy. The proton beam spectrum shown in Fig. 5 suggests that fusion is about a factor 100–1000 favored over ^11^C production. However, non-laser, non-plasma beam stopping power wisdom suggests that primary reactions dominate secondary by a factor 10,000. In addition we use ^18^F as diagnostic, which is suppressed by its electromagnetic production process, thus one can easily argue that one ^18^F is suppressed by a factor million, and this suppression is reduced to 1000–10,000 by the structure of the primary proton beam. Should ^18^F at a higher yield than ^11^C/1000 be observed, this signals that secondary reactions are enhanced by plasma environment. We conceived this experiment in order to establish the strength of this effect.

The second and equally important point is the observation of the modification of the ^11^C production in BN plasma as compared to B plasma. This result means that, beyond the plasma action, new effects occur that we attributed to the reaction cycles induced by the secondary reactions described above. It appears that in the plasma state the reaction rates are increased and this also applies to the secondary reactions. The cycle of reactions ^11^B(p,α)2α followed by ^14^N(α,p)^17^O reiterates the reactions with ^11^B, including the reaction ^11^B(p,n)^11^C producing the additional ^11^C. This hypothesis also explains the increased number of α-particles in the case of BN compared to B targets. The effect of the time delay between the two laser pulses further demonstrates that the temperature and the ionization state of the target affects the nuclear reaction rates. In Fig. 6b, the time of arrival of the protons in the plasma as a function of their energy is shown for the three time-delays. For the case, −100 ps, all the protons of interesting energy (>500 keV) interact with a hot plasma. For the case, +200 ps, the protons with energy below 700 keV arrive after the end of the plasma formation pulse and in a plasma with a lower temperature than in the previous case. For the case +500 ps, all the protons arrive after the end of the plasma formation. This means that the plasma entered the recombination phase with a temperature which decreases rapidly. The results are nevertheless different from those of the solid state. All this is consistent with and explains the observed decrease of A(BN)/A(B) as a function of increasing time delay.

## Conclusion

In summary, our results demonstrate that it is possible to produce a charged particle chain of reactions using boron-nitride targets irradiated by a laser-accelerated proton beam. We believe that this result is an important stepping stone towards energy production by laser fusion as well as to the production of required yields of medical radio-isotopes by laser. Our experimental conditions were not optimized for the ^18^F and ^11^C productions, which explains the relatively low yields that are observed. Our results demonstrate the proof of principle that i) secondary reactions with the α-particle are possible in the target and ii) that the rates of the desired reactions can be significantly increased by the chain of reactions. In the future, improvements can be expected by optimization of isotope mixture, of the proton spectra (as was observed with gold), of the plasma formation and of the target characteristics.

## Methods

### Experimental set-up and laser-accelerated proton beam

Experiments were conducted on the Elfie laser facility at Ecole Polytechnique. The scheme of the experimental set up is shown in Fig. 1. The short laser pulse, with 10 J in 0.35 ps at wavelength 1.06 μm, was focused with an off-axis parabolic mirror at normal incidence on a thin foil at an intensity of 1–2 × 10^19 ^W.cm^−2^, to produce a proton beam by the Target Normal Sheath Acceleration (TNSA) mechanism^7^,^8^. We used three types of target: Aluminum of 10 and 20 μm thickness and Gold with 10 μm thickness. The energy spectrum of the proton beam was characterized using Thomson parabola and CR39 track-detector covered with Aluminum foils of different thickness to select the proton energy ranges. The integrated proton spectra for the three types of target are shown in Fig. 5 for two laser intensities. We observe that the low-energy part of the spectrum is similar for Al-20 μm and Au-10 μm. It is reduced for Al-10 μm or for lower short pulse intensity. The high-energy cutoff is larger for gold (~6.8 ± 0.5 MeV) than for Aluminum of 20 μm (~5.8 ± 0.5 MeV) and Aluminum of 10 μm (3.3 ± 0.5 MeV). This is in agreement with the CR39 measurements as well as with previous studies of TNSA proton acceleration. The integrated number of protons rises from 10^9 ^sr^−1^ at high energy to ~10^13 ^sr^−1^ at low energy. With reduced laser intensity, the energy cut-off is lowered and the integrated number of protons significantly reduced.

The proton beam was sent on a boron (B) or boron-nitride (BN) thick foil (500 μm-1 mm) placed at 20° from the proton axis at a distance of 6 mm from the proton target. In both cases, natural Boron was used, including the two isotopes ^10^B and ^11^B. For part of the shots, the proton beam interacted directly with the target initially in the solid state and, for the other part, the target was previously turned into plasma by the nanosecond pulse. This pulse delivered 80J in 0.6 ns at 1.06 μm leading to an intensity of 10^13 ^W/cm^2^ on target. The short and the long pulses come from the same oscillator so a perfect synchronization was easily provided and different time-delays between the two laser pulses were used. The electron temperature of this plasma was ~0.3–0.5 keV.

### Diagnostics

Particle diagnostics included a magnetic spectrometer, two Thomson parabolas and CR39 track-detectors. In purpose, the magnetic spectrometer and the Thomson parabola were set to collect α-particles in different ranges of energy, high- and low-energy ranges respectively. The diagnostics were installed in the horizontal plane at different locations as shown in Fig. 1. The CR39 were previously calibrated in the laboratory^23^,^24^ and were used to distinguish the protons from the alpha particles by the size of the impact diameter, ~(1 ± 0.5)μm for protons and ~(2.8 ± 0.5)μm for α-particles. However, all these diagnostic led to an underestimation of the number of particles because the only ones that can be detected are those that come out of the target^25^. So, to complete these data, we implemented activation measurements of the target after the shot as a function of time with a Geiger-Mueller tube detector with a precision of ±5%. The activation measurements allow the determination of the amount of isotope formed in the target and so provide the number of the studied reactions without depending on the output capacity of the particles off the target.

### Objective and principle of the method

The objective of these experiments is to launch secondary nuclear reactions by a clever choice of complementary components of the target irradiated by the proton beam. The advantage of secondary reactions is the production of the elements that are needed to repeat the primary reactions and so to increase their yield. In the case of our experiments, the α-particles produced in the primary reactions react either with ^14^N or ^10^B to generate ‘new’ protons that have the right energy to react again with the ^11^B. The other secondary reactions are used as diagnostic tool. The observation of ^18^F unequivocally demonstrates that α-particles have reacted with ^14^N. This is a unique signature. In our experiments, the unique feature of the secondary reactions is that they use particles that are produced *in situ* in the target whereas the primary reactions are produced by the accelerated proton beam coming from the Aluminum foil. The observation of such reactions is of great interest for many applications where it can be cheaper to produce the reactant by interaction with a target rather than accelerating the ions.

## Additional Information

**How to cite this article**: Labaune, C. *et al.* Laser-initiated primary and secondary nuclear reactions in Boron-Nitride. *Sci. Rep.* **6**, 21202; doi: 10.1038/srep21202 (2016).
